# Galactose tolerance in adults with classical galactosaemia. Considering the gaps

**DOI:** 10.1016/j.ymgmr.2026.101298

**Published:** 2026-02-26

**Authors:** L.A. Shakerdi, C. Newman Thacker, K. Moore, A. Sheerin, M. Noga, M.E. Rubio-Gozalbo, G.T. Berry, J.J. O'Byrne, R. Saldova, E.P. Treacy

**Affiliations:** a National Centre for Inherited Metabolic Disorders, The Mater Misericordiae University Hospital, Dublin, Ireland; b School of Public Health, University College Dublin, Dublin, Ireland; c Mosakids Childrens Hospital/Laboratory of Clinical Genetics, Maastricht University Medical Centre, Member of MetabERN and member of United for Metabolic Diseases, Grow Research Institute, Maastricht University, Maastricht, the Netherlands; d Division of Genetics and Genomics, Boston Children's Hospital, Harvard Medical School, Manton Centre for Orphan Disease Research, Boston, MA, United States; e UCD School of Medicine, College of Health and Agricultural Science (CHAS), University College Dublin (UCD), Dublin, Ireland; f School of Medicine, Trinity College Dublin, Dublin, Ireland; g NIBRT GlycoScience Group, National Institute for Bioprocessing, Research and Training (NIBRT), Dublin, Ireland; h CÚRAM, SFI Research Centre for Medical Devices, National University of Ireland, Galway, Ireland

**Keywords:** Classical galactosaemia, Adults, Galactose-1-phosphate, Dietary galactose, Immunoglobulin G, *N*-glycosylation

## Abstract

Classical galactosaemia (CG, OMIM 230400) is a rare inborn error of metabolism caused by deficiency of galactose-1-phosphate uridylyltransferase. The available modality of treatment, a galactose-restricted diet, is effective in preventing life-threatening neonatal symptoms. However long-term complications including neurological, speech and fertility issues in females remain prevalent.

**Methods:**

This retrospective review reports the experience of mild-moderate relaxation of dietary galactose intake over time in a cohort of 31 Irish CG adult patients with established RBC Gal-1-P, and novel IgG *N*-glycan analysis. Three groups were categorised based on the retrospective analysis of estimated dietary galactose intake: Group 1 (<200 mg/day), Group 2 (200–500 mg/day) and Group 3 (501-1000 mg/day). Dietary galactose intake was compared to matching RBC Gal-1-P levels and serum IgG *N-*glycan profiles (measured by HILIC-UPLC).

**Results:**

RBC Gal-1-P levels increased with increased galactose intake with statistically significant differences only between the lowest and highest galactose intake group (*p* < 0.05). Minor changes were seen in a number of IgG *N*-glycans in the highest galactose intake group with increases in core fucosylated monoantennary and biantennary monogalactosylated monosialylated glycans, pentamannosylated glycans, oligomannosylated glycans and monoantennary glycans with a decrease in grouped biantennary glycans. The most significant change noted was an increase in pentamannosylated glycans with increased dietary galactose intake.

**Conclusion:**

Moderate relaxation of dietary galactose intake (up to 500 mg/day) may be well tolerated in the majority of CG adults. These data suggest that serum *N*-glycan profiling may provide an improved individualised ‘personalised medicine’ approach for treatment interventions for CG, considering individualised variation in glycosylation, including ‘glycosylation outliers’.

## 1 Introduction

Classical galactosaemia (CG, OMIM 230400) is a rare inborn error of carbohydrate metabolism, caused by a severe deficiency of the enzyme galactose-1-phosphate uridylyltransferase (GALT E.C. 2.7.7.12 [1], [2]. A galactose-restricted diet has proven to be very effective to treat the neonatal life-threatening manifestations. This prevents often fatal liver disease and other immediate complications. However, despite early treatment the majority of affected patients go on to develop one or more long-term complications, including cognitive deficits, speech and language abnormalities and delay, neurological and fine motor impairments, and primary ovarian insufficiency in females [1], [2], [3], [4], [5], [6], [7]. These complications are well documented in the international Galactosaemia Network (GalNet) outcome registry and historically in a number of outcomes studies [1], [3], [4], [5], [6], [7]. Although homozygosity for the common CG disease causing variant (c.563 A > G, p.Gln188Arg) in Caucasian populations has been reported to independently associate with poorer outcomes, there does not appear to be a robust established correlation between the occurrence of long term complications with the rigor of dietary galactose restriction [1], [7], [8], [9], [10]. An outcome study in the US of 231 children and young adults with CG which compared the rigor of strict and slightly relaxed diet (including non-dairy sources of galactose such as legumes and some fruits (up to 100 mg/day)), demonstrated no significant differences of moderate galactose liberalisation in the outcomes measured (receipt of speech therapy, need for educational supports, markers of ovarian function) [8]. The GalNet Galactosemias registry also recorded observational data on 509 CG subjects derived from 15 countries and 32 centres [1]. Those on a strict diet (lactose restricted and restrictions in fruit and vegetables) developed neurological complications more frequently (*p* < 0.001; OR 2.81 [1.64–4.50]) than those with a less strict diet.

Historically, since the clinical description of CG, the treatment has focussed on strict restriction of all known sources of galactose from the diet, including galactose in nucleoproteins, fruit and vegetables (galactosides). The original UK 1992 recommendations on treatment of CG advised severe restriction of galactose (from dairy and non-dairy sources including galactosides, fruit and vegetables), and recommended a galactose intake of <20 mg/day [11]. In the first international CG clinical guideline, following satisfactory experience in a number of countries, a core recommendation was to allow any amount and type of fruits, vegetables, legumes, unfermented soy-based products, mature cheeses (with galactose content <25 mg/100 g), and the food additives sodium or calcium caseinate, which could account for a predicted dietary intake of maximum of 200 mg/galactose intake per day [12]. This first CG guideline also stated that “there was insufficient evidence to support a specific age-related recommendation for the quantity of galactose allowed in the diet”.

As noted in the literature, adult patients with CG have previously been reported to relax the galactose restriction without adverse events noted for moderate relaxation [13], [14]. It is also debated how stringent diet should be after infancy, as endogenous galactose production is higher than that ingested from foods other than dairy products [15], [16]. An earlier clinical trial of a moderate galactose relaxation of up to 500 mg in children was well tolerated [17].

Previously, US practice recommended that dietary restrictions on all lactose-containing foods including cow's milk and other dairy products should continue throughout life [18]. However, in the US, more recent dietary recommendations allow all fruits, vegetables, legumes, soy products that are not fermented, various aged cheeses and foods containing caseinates [19], which is consistent with the international Galactosaemia guideline [12].

The biochemical consequences of impaired galactose metabolism are abnormally high levels of galactose and its derivatives in body tissues and fluids. Galactose metabolites, which are measured in tissues such as RBC Gal-1-P and urinary or plasma galactitol, have been used to monitor dietary compliance in children, adolescents and adults with CG. Prior to treatment in the newborn, RBC Gal-1-P levels in the neonate may be as high as 2.5 to 6 mMol/L in packed blood cells [2]. Following dietary restriction of galactose, the levels fall dramatically. On follow up of cohorts of CG children (UK and USA) who were historically treated with strict galactose restriction, it has been recommended to date that the upper limit of Gal-1-P in RBCs that is considered to be acceptable is 150 μmol/l in RBCs (equivalent to 5 mg/dl or 0.5 μmol/gHb) [11], [18].

Hutchesson and colleagues concluded that high intraindividual variability for Gal-1-P may limit its utility for biochemical monitoring [20]. It has been reported that, although RBC Gal-1-P monitoring may identify major galactose intoxication and significant dietary non-adherance in CG, this may not identify moderate differences in galactose intake in individuals with CG [8], [9], [10]. In terms of glycosylation as a variable feature in CG, abnormal glycosylation as a secondary glycosylation defect is well documented in CG [21], [22], [23]. In a number of studies our group has identified ongoing *N*-glycan processing abnormalities in dietary treated CG children and adults [21], [24], [25], [26], [27]. Milder *O*-glycosylation defects have also been observed post dietary treatment in CG individuals [28].

Previous studies by our group in adult CG individuals looked at galactose dietary increments of up to 4000 mg/day over a 16 week period and indicated there was no marked difference in RBC Gal-1-P levels or urinary galactitol levels, liver function tests and eye examinations (for cataract development), in these individuals for a galactose intake of up to 1000 mg/day during the study period [24]. Moderate galactose intake correlated positively with improvements in monosialylated, monogalactosylated, and monoantennary *N*-glycan structures, as measured by a novel IgG *N*-glycan glycome HILIC analytical platform. In a follow up paediatric study, thirteen CG children were provided with 300 mg of galactose/day followed by 500 mg for 2 weeks each (with 13 matched patient controls) [25]. There were no clinical changes seen with the intervention. A temporary mild increase in RBC Gal-1-P levels occurred in one individual, but renal, liver, and bone biochemistry analyses remained normal. A number of individuals showed an improved glycosylation pattern with the increased galactose intake.

It is generally now considered, with possible exceptions, that CG is not a progressive condition in adults [29]. However, the significance of persistent mild glycosylation abnormalities observed in CG adults (noted after completion of brain myelination) remains unclear. For example, in the context of brain glycolipid formation which could be affected in CG, galactocerebroside and its derivative sulfatide is a major component of the myelin sheath and myelin integrity is critical for the rapid transmission of nerve impulses [30].

Neurological and developmental outcomes measured to adult life can vary even among siblings with the same GALT genotype, highlighting the complexity of this condition [9], [31]. This complexity is probably influenced, for example, by significant epigenetic effects, identified common glycan polymorphic genes, intricate accessory glycosylation pathways with significant variation noted in glycosylation in healthy controls, differences in inflammatory responses, and cellular stress events [32], [33]. For example, the glycome of immunoglobulins is noted to be highly variable with high heritability with polymorphisms of the glycan genes encoding the glycosyltransferases ST6GAL1, B4GALT1, FUT8, and MGAT3, noted to represent the most important loci associated with variation in IgG traits [33], [34].

As stated above, historically, the approach to treatment of CG has been to restrict all exogenous galactose food sources. Concomitantly, the optimum reference range for those on strict galactose restriction was based on means obtained from the monitoring of adherent CG individuals on strict dietary galactose restriction. In contrast to the example of hyperphenylalaninaemia for neurocognitive outcomes, there is absence of data correlating the optimum levels of RBC Gal-1-P with long term neurological or fertility outcomes in females [35]. Also, there is a lack of long-term outcome studies of mild to moderate galactose liberalisation in CG adults.

In this report we reviewed retrospectively the longer-term effects of mild dietary galactose liberalisation in the Irish adult CG cohort with the use of routine biochemical and IgG *N*-glycan monitoring.

## 2 Materials and methods

### 2.1 Patient characteristics

A total of 31 adult classical galactosaemia (CG) patients had matched RBC Gal-1-P levels, serum IgG *N*-glycosylation profiles, blood liver function tests, routine opthamological reviews and dietary recall undertaken at routine clinic visits according to scheduled clinical monitoring. All patients have classical galactosaemia according to their genotypes. All were followed at the National Centre for Inherited Metabolic Disorders adult site at Mater Misericordiae University Hospital, Dublin (Table 1).

**Table 1:** Characteristics of CG patients

Subjects (n)	31
Age range (years; Mean ± SD)	36.3 ± 10.4
Gender	14 Males, 17 Females
*GALT* genotype	28: p.Gln188Arg/p.Gln188Arg
	1: p.Gln188Arg/p.Arg333Trp
	1: p.Gln188Arg/p.Lys127Glu
	1: p.Gln188Arg/p.Phe194Leu
FSIQ* (n= 23)	Mean: 85, Range 52-126

FSIQ* (as available). Full-Scale Intelligence Quotient. 90-109, average 25^th^ to 74^th^, represents the average range of intellectual functioning.

The most recent FSIQ assessment performed for patients when available (using standardized psychological testing) as documented in the patients' case notes) was noted. The standardized tests used were either the Wechsler Intelligence Scale for Children (WISC) or the Wechsler Adult Intelligence Scale (WAIS) according to the age at testing.

The clinical laboratory testing and documented dietary analysis period for this retrospective review was during the years 2022–2023. The dietary galactose intake was estimated retrospectively by analysing the dietary recall taken at the routine clinic visit when the specific blood monitoring was undertaken [36]. Galactose containing foods were identified (not including ‘bound galactose’). In this context the main source of galactose in the human diet is **lactose**, the primary sugar found in milk and dairy products. In addition, many manufactured foods (including biscuits, processed meats, breads and sauces) contain hidden lactose/galactose in small amounts. Small amounts of free or bound galactose also exist in certain fruits and vegetables (free and bound galactose in galactolipids and glycoproteins), legumes and beans (galactosides and oligosaccharides), offal and eggs.

The galactose content of these foods was calculated using the analysis of the galactose content of cheeses and the detailed galactose analysis of commonly preferred foods requested by our patient cohort, as we reported previously [37]. For foods that were of a different brand to the brands previously analysed, the estimated galactose intake of the most similar product previously analysed was used. Each patient's total galactose intake per day (mg/day) was calculated.

The majority of the individuals who followed a galactose intake over 200 mg/day (*n* = 7) had been on this amount of galactose for at least one year at the time of blood sampling.

According to the practice at the time of this profiling, CG adults attending the clinic were offered to follow a dietary galactose restriction of less than 200 mg/day, or a relaxation to 200–500 mg galactose/day, with monitoring.

### 2.2 Measurement of RBC Gal-1-P

RBC Gal-1-P was measured by mass spectrometry at Bristol University Hospital (UKAS accredited) as previously described [38].

### 2.3 Processing of collected blood samples for IgG *N*-glycan analysis

The IgG isolation was carried out from prepared serum samples using a Protein G Spin Plate for IgG Screening from Thermo Scientific, as per manufacturer's instructions. *N*-glycans were released from the isolated IgG using the high-throughput hydrophilic interaction ultra-performance liquid chromatography (HILIC-UPLC) method previously described [39]. A control of pooled serum of normal healthy adults purchased from Sigma Aldrich was used. This serum is pooled from multiple donors, healthy males and females from 400 to 500 donors, age range 18 to 65 years, sourced in the US from FDA licensed facilities.

**Fig. 1:**
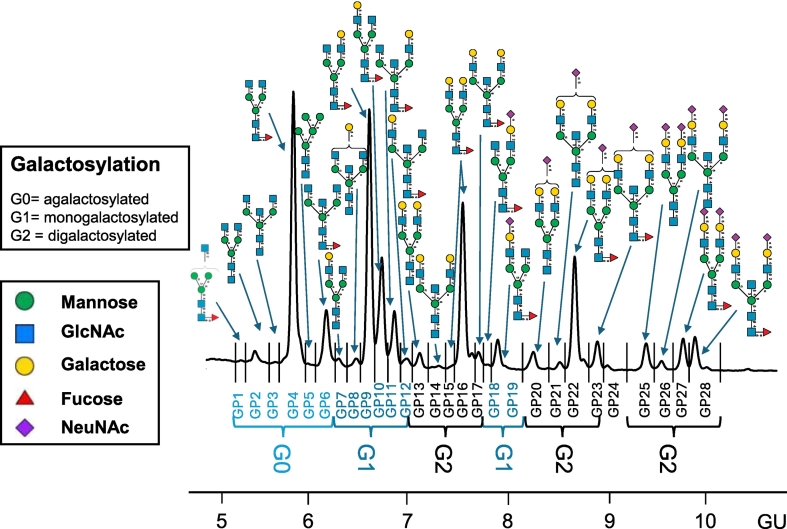
IgG *N*-glycans, pictured are the major glycans and highlighted galactosylation in the healthy control sample. Legend for glycan peaks: Monoantennary (MA, GP1) and biantennary (BA, GP2–4 + GP6–23 + GP25–28) complex glycans, or oligomannosylated glycans (OM, GP5/2), galactosylation (agalactosylated (G0, GP1–6), monogalactosylated (G1, GP7–12 + GP18–19) and digalactosylated (G2, GP13–17 + GP20–23 + GP25–28) structures), sialylation (asialyated (S0, GP1–17), monosialyated (S1, GP18–23) glycans), disialylated (S2, GP25-28), fucosylation (core-fucosylated (CF, GP1 + GP4 + GP6 + GP9–12 + GP15–19 + GP22–23 + GP27–28) glycans) and bisecting glycans (B, GP3 + GP6 + GP8 + GP11–12 + GP14 + GP17 + GP21 + GP23 + GP26 + GP28). Core fucosylated neutral glycans (Fn, GP1 + GP4 + GP9–10 + GP16), core fucosylated bisected neutral glycans (FBn, GP6 + GP11–12 + GP17) and afucosylated bisected neutral glycans (Bn, GP3 + GP8 + GP14).

The isolated *N*-glycans included twenty-eight *N*-glycan peaks (GP), previously characterised by UPLC and identified by mass spectrometry identified (Fig. 1). The glycan groups were grouped according to common features as shown in the legend to Fig. 1: branching (monoantennary- (MA), biantennary (BA) complex glycans or oligomannosylated (OM), galactosylation (agalactosylated (G0), monogalactosylated (G1) and digalactosylated (G2), sialylation (asialyated (S0), monosialyated (S1), fucosylation (core-fucosylated: CF), bisecting glycans (B). Other specific features were also investigated: core fucosylated neutral glycans (Fn), core fucosylated bisected neutral glycans (FBn), and afucosylated bisected neutral glycans. Grouped glycan features were also analysed as above including galactosylation, sialylation, fucosylation, and bisecting glycans [34], [40]. The percentage areas of the peaks for agalactosylated (G0), monogalactosylated (G1), and digalactosylated (G2) structures were measured to quantitatively assess the galactose incorporation into IgG *N*-glycans, as previously outlined [41].

### 2.4 Statistical analysis

IBM SPSS Statistics version 29.0.1.0 (171) (SPSS Inc., Chicago, Illinois) and R studio (version 2024.04.2 + 764) were used for statistical analyses. Data normality was performed using Lilliefors-corrected Kolmogorov-Smirnov test and equality of variances were tested using Levene's test for homogeneity of variances. Based on these results, parametric and nonparametric tests were used. For the clinical data, parametric tests the ANOVA test with Tukey's adjustment for multiple group testing were used for normally distributed and homogenous data, and for non-parametric tests Kruskal Wallis with Dunn's adjustments for multiple group analyses were used on data which were not normally distributed and/or with non-homogenous variances. Glycan data (which were mainly normally distributed and had mainly homogenous variances) were logit transformed for more normal distribution, and parametric tests (ANOVA with Tukey's adjustment for multiple group analyses) with Benjamini Hochberg (BH) correction for multiple variables were used (for multiple related glycan peaks (GPs), and features) [40]. All data, clinical variable and glycans (individual peaks and groups), were correlated using non-parametric Spearman tests due to mixed data nature regarding normality and homogeneity of variances. Visualization was performed in Python associated packages Seaborn and Matplotlib. Statistical significance was considered at *p* < 0.05. As the patients were unequally distributed in the groups additional statistical analyses for small effect sizes using omega squared, (which is particularly suitable for small sample sizes) were performed not to only assess the *p*-values which could be falsely not significant, but also importance by effect sizes [42], [43]. A Partial Least Squares Discriminant Analysis statistical analysis (PLS-DA) was also performed using Python associated package sklearn. This is a statistical method used for classification of different groups of subjects (often patients vs controls). The assigned Variable Importance in Projection (VIP) score measures a variable's importance in the PLS-DA plot. Generally, VIP scores of 1 or greater are considered important for the model. Data fed into the model were standardized using preprocessing scale function to fit into the PCA model (all features are on the same relative scale).

## 3 Results

### 3.1 Patient characteristics

The characteristics of the 31 adult CG participants are shown in Table 1. Three dietary groups were noted according to estimated dietary galactose intake. Group 1, intake of <200 mg galactose/day, (*n* = 23) Group 2, 200–500 mg/day (*n* = 4), and Group 3, 501-1000 mg/day (*n* = 3) (Table 2). Thus, the majority of the cohort elected to maintain the more stringent daily dietary galactose intake. Of note, there was one additional individual who was non-adherent, with significant deviations from the galactose restricted dietary advice (estimated galactose intake>2000 mg-2500 mg galactose/day). This individual was not included in the IgG *N*-glycan group statistical analysis because of (*n* = 1).

**Table 2:** CG individual groups according to Galactose daily dietary intake.*

Group	Daily Intake of Galactose	Male	Female	Male + Female
1	<200mg	10	13	23
2	200 – 500mg	1	3	4
3	501 – 1000mg	3		3
Total		14	16	30

*Individual with intake> 1,000mg not included in group analysis as n=1.

There were no changes noted in LFTS or routine eye examinations over the study time period for all individuals. The overall mean and FSIQ ranges available for the cohort are shown in Table 1.

**Fig. 2:**
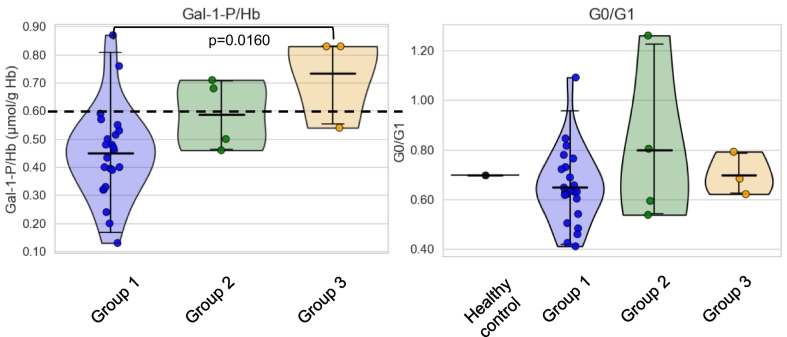
Gal-1-P levels and G0/G1 in the study groups. Group 1: <200 mg gal/day, Group 2: 200–500 mg/day, Group 3: 500–1000 mg/day. Violin plots were created using Seaborn package, 95% confidence interval with the mean value indicated as straight horizontal bars. ANOVA with Tukey HSD was used for *p*-values calculations. Significant adjusted *p* values are labelled. The dotted line represents the upper limit of the reference laboratory recommended CG optimum treatment range for RBC Gal-1-P levels. For the G0/G1 ratios, the pooled healthy control value is indicated (0.70).

**Fig. 3:**
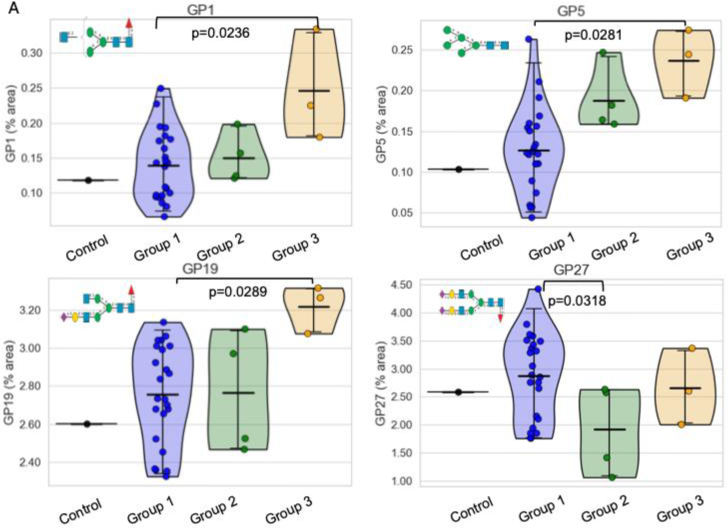

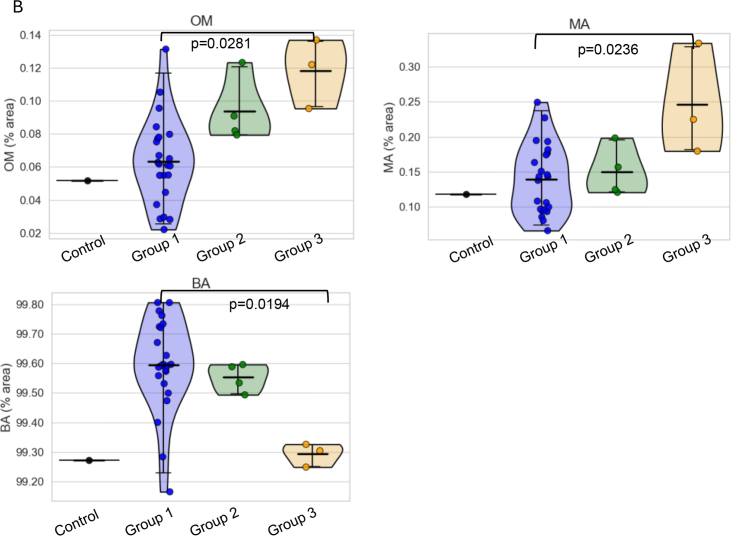
Selected A) GPs and B) Glycan feature levels in the study groups. Group 1: <200 mg gal/day, Group 2: 200–500 mg, Group 3: 500–1000 mg. Sns violin plots were created using Seaborn package, 95% confidence interval with the mean value indicated. ANOVA with Tukey HSD was used for p-values calculations. Significant adjusted p values are labelled (without correction for multiple peaks/features), and the major glycans in each GP are pictured. Legend for glycans pictured: GP1: FA1, GP5: Mainly M5, GP19: FA2[3]G1S(6)1, GP27: FA2G2S(6,6)2, OM: Oligomannosylated, MA: Monoantennary, BA: Biantennary.

Table 3 Illustrates a summary of the relevant significant findings for RBC Gal-1-P levels and specific glycans in comparison to the pooled healthy control, which are further illustrated in Fig. 2, Fig. 3. Fig. 1 illustrates the *N*-glycans from the control IgG pooled sample as analysed by HILIC-UPLC, with highlighted galactosylation features.

**Table 3:** Gal-1-P levels and significant glycans.^#^

Clinical and glycan features	Gal-1-P/Hb	Age	GP1	GP5	GP19
		% area			
	Mean ± SD				
Group 1 (n: 23)	0.45 ± 0.17	33.65 ± 9.34	0.14 ± 0.05	0.13 ± 0.05	2.75 ± 0.26
Group 2 (n: 4)	0.59 ± 0.13	51.25 ± 7.80	0.15 ± 0.04	0.19 ± 0.04	2.77 ± 0.32
Group 3 (n: 3)	0.73 ± 0.17	39.00 ± 1.73	0.25 ± 0.08	0.24 ± 0.04	3.22 ± 0.13
All groups (n: 30)	0.50 ± 0.18	36.53 ± 10.47	0.15 ± 0.06	0.15 ± 0.06	2.80 ± 0.29
Control	<0.60 μmol/g Hb*	18-65	0.12	0.10	2.60


Clinical and glycan features	OM	MA	BA	G0/G1
Group 1 (n: 23)	0.06 ± 0.03	0.14 ± 0.05	99.59 ± 0.16	0.65 ± 0.15
Group 2 (n: 4)	0.09 ± 0.02	0.15 ± 0.04	99.55 ± 0.05	0.80 ± 0.33
Group 3 (n: 4)	0.12 ± 0.02	0.25 ± 0.08	99.29 ± 0.04	0.70 ± 0.09
All groups (n: 30)	0.07 ± 0.03	0.15 ± 0.06	99.56 ± 0.17	0.67 ± 0.18
Control	0.05	0.12	99.27	0.70

*Bristol University Hospital reference laboratory acceptable level for a galactosaemic on a galactose restricted diet: <0.60 μmol/g Hb.

^#^Significant differences are illustrated in Fig. 2, Fig. 3.

*Individual with intake> 1,000mg not included in group analysis as n = 1.

RBC Gal-1-P levels were found to be significantly different between the lowest galactose intake group (group 1) and the highest galactose intake group (group 3), (Table 3 and Fig. 2) (*p* = 0.016). The G0/G1 ratios were not statistically different between the groups. As age is known to affect glycosylation, age as a confounding variable was assessed [44]. Age as a variable did not cause noticeable differences in our cohort (Supplementary Table S1).

Several glycan peaks (GPs) and features were significantly changed with galactose intake (see Fig. 3), namely, glycan peaks GP1 (core fucosylated monoantennary glycans), GP5 (mainly pentamannosylated glycans), and GP19 (mainly core fucosylated biantennary monogalactosylated monosialylated glycans) all showing significant increases in groups 3 from group 1. For the grouped glycan features, group 3 also showed higher OM features (oligomannosylated glycans), higher MA (branching monoantennary) and lower BA features (biantennary) groups (all *p* < 0.05). This significance, however, was lost with further correction for multiple testing of GPs and features, which is not surprising given that the groups 2 and 3 have very few patients. Therefore, effect sizes were examined to check potential importance in separating groups, and they were all large, with the highest value noted for GP5 (mainly pentamannosylated glycans) indicating that all the changes in the GPs and features are important. Low GP5 have been shown to be highly significant in our previous studies in CG involving different populations with also lower GPs 1, 5, OM and MA and MA [7], [21], [27].

Of note, there were no statistically significant difference in overall fucosylation, galactosylation and sialyation features with higher galactose intakes (as previously noted as predominant abnormal features between CG patients and controls, Supplementary Figure S1).

**Fig. 4:**
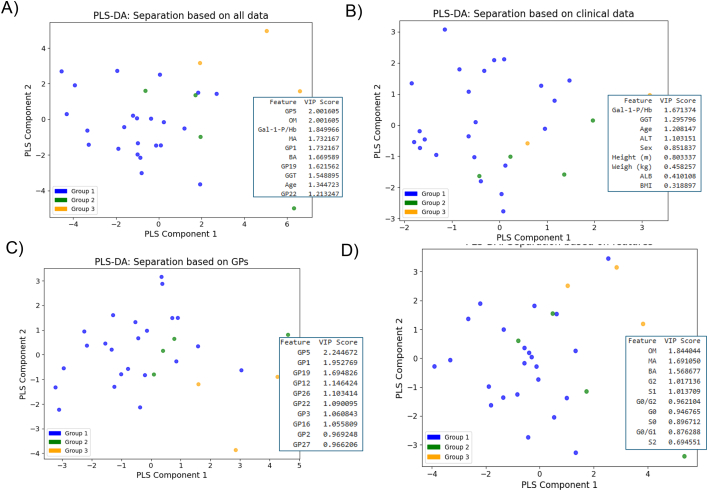
PLS-DA plots to separate dietary groups, based on inclusion of: A) RBC Gal-1-P levels, age and main glycan peaks and features B) RBC Gal-1-P levels and clinical variable parameters only, C) only glycan GPs and D) only grouped glycan features. Each patient in each group is represented with a specific colour to the group. The VIP score shows the 10 most important features for each separation.

Fig. 4 illustrates the separation of 3 dietary groups using PLS-DA plots, based on age, dietary galactose intake groupings, RBC Gal-1-P levels, specific glycan data and their combination. Subjects from group 3 are seen to cluster together and are separated from subjects from other groups in the plots (Fig. 4). Important features in these separations confirm the importance of statistically significant Gal-1-P levels and the significant GPs and features described above (VIP > 1) with GP5 being the most significant variable of all the noted features.

**Fig. 5:**
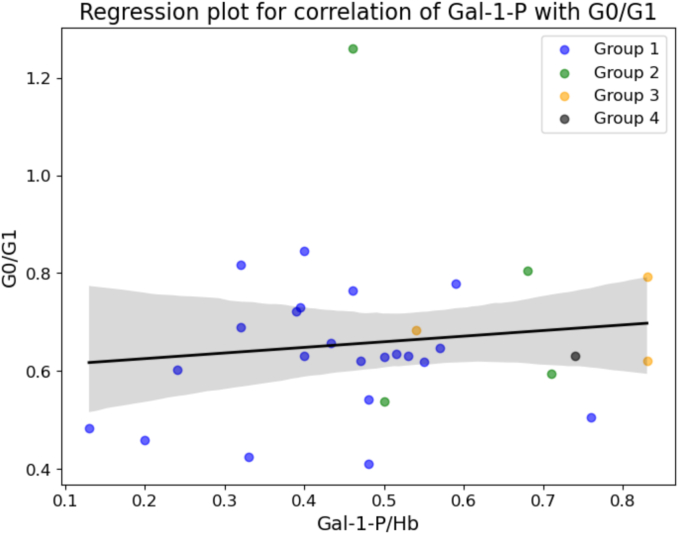
Regression plot for Gal-1P and G0/G1 levels. Spearman rho = 0.0695, *p* = 0.7153. Note: The 3 groups are depicted. Group 4 has only one individual/data point illustrating the individual with an estimated dietary galactose intake>2000 mg/day.

Comparing the RBC Gal-1-P levels with G0/G1 ratios, a positive correlation was identified although not statistically significant (Spearman rho = 0.0695, *p* = 0.7153), which may be explained by the small sample size and a number of outliers in Group 1 who demonstrated more pronounced abnormalities of *N*-glycosylation (Fig. 5). These individuals (*n* = 3) have optimum RBC Gal-1-P levels (< 0.6 μmol/gHb), however, at the time of testing show markedly elevated G0/G1 ratios (outside the group standard deviation), and higher than the control. These outliers potentially confound the correlation between RBC Gal-1-P and G0/G1 ratios perhaps illustrating epigenetic effects or differences in accessory galactose pathway utilisation as we have previously noted with individual differences in galactose tolerance. The higher dietary galactose intake groups (in particular group 3), however, show a trend towards higher values of both Gal-1-P levels and G0/G1 ratios (Fig. 5).

## 4 Discussion

An important question, which remains to be addressed in classical galactosaemia is the consideration of moderate galactose liberalisation in CG adults, which might improve quality of life without changing immediate or long-term outcomes. In addition to this, is the reconsideration of galactose tolerance using current RBC Gal-1-P threshold levels as a measurable clinical end point for emerging new therapies for Classical Galactosaemia. This questions the current rationale for the optimum reference range of monitoring of RBC Gal-1-P levels, and also the necessity for more personalised monitoring biomarkers as in the example of known variability in individualised *N*-glycosylation profiles.

Specifically the commonest disease-causing *GALT* variant in CG, p.Gln188Arg has been identified as a potential factor influencing the severity of long-term outcomes. However, variability in outcomes have been consistently shown even in siblings with identical genotypes and dietary galactose intake, suggesting the presence of significant genetic/epigenetic modifiers [32].

In this retrospective review, we have noted that RBC Gal-1-P levels were well-controlled with a galactose intake of less than 200 mg/day and were also within the laboratory CG recommended range of <0.6 μmol/Hb with galactose intakes levels between 200 and 500 mg/day. These findings are also in conjunction with normal routine clinical monitoring (LFTs, eye reviews) in all monitored patients. These observations imply that moderate increases in galactose intake are generally well tolerated by adult CG patients and do not result in significant alterations in RBC Gal-1-P levels. However, in group 3, RBC Gal-1-P levels were significantly higher than for the reference laboratory recommended range and were significantly higher than for group 1.

Galactosemia is associated with abnormalities in *N*-glycosylation, which includes both processing and assembly defects [21], [22], [23]. Sturiale et al. previously reported in untreated galactosaemia a partial deficiency of whole *N*-glycans in serum transferrin associated with increased fucosylation and branching as seen in Congenital Disorder of Glycosylation (CDG) Type 1. This, and other reports suggest that there are joint *N*-glycan assembly and processing defects in untreated galactosaemia [23], [45], [46]. *N*-glycosylation assembly defects are clearly present in CG neonates before introduction of diet and processing defects are well described to persist in CG diet treated children and adults [24], [26], [31], [45], [46].

In addition to the well characterised, extreme life-threatening neonatal phenotype of untreated classical galactosaemia, there are reports of significant developmental delay/intellectual disability in late diagnosed (untreated) adult classical galactosaemia individuals [47]. Interestingly, Quelhas and colleagues described three young adults with late identified and treated CG (with undetectable or minimum detectable RBC GALT activity). Prior to galactose dietary restriction, two of these untreated individuals were shown to have a CDG Type 1 transferrin IEF pattern and the third individual demonstrated a CDG Type 2 transferrin IEF pattern [47].

IgG, the most predominant circulating glycoprotein plays an important role in the human immune system. IgG has modifiable *N*-glycans attached to the Fc region which can switch its functionality. The absence of sialic acid changes the physiological role of IgG from anti-inflammatory to pro-inflammatory and increased IgG fucosylation is associated with the severity of rheumatoid arthritis [48], [49], [50].

In this report, it was noted that the glycan groups GP1 (containing core fucosylated monoantennary glycans), GP5 (mainly pentamannosylated glycans) and GP19 (mainly core fucosylated biantennary monogalactosylated monosialylated glycans) were increased in group 3 versus group 1. For the grouped glycan features, group 3 also showed higher OM features (complex glycans or oligomannosylated), higher MA (branching monoantennary) and lower BA features (biantennary) groups (all *p*< 0.05). The significance of these minor differences is unclear in the context of very small subject numbers in the dietary groups 2 and 3 and could be explained by individual subject variation.

In previous work involving different country CG cohorts using IgG, we have confirmed decreased *N*-galactosylation and increased fucosylation in CG as compared to controls [21], [27]. However, in this current study, although we noted minor *N*-glycosylation changes of unknown long term significance, there was no notable differences in the three different CG dietary groups (with galactose intake of up to 1000 mg/day) for *N*-galactosylation or fucosylation. This suggests that mild-moderate galactose liberalisation may be well tolerated in CG adults without long term clinical adverse outcomes. The most significant change in glycan features noted among the groups however was the increase of mannose 5 (Man5)  glycans, GP5 with increased galactose intake in Group 3. A decrease in this feature was found to be highly significant in our previous studies in differing population CG cohorts [7], [21], [27].

A critical step in the maturation pathway in Golgi processing of *N*-glycans is the addition of galactose (Gal) to the core *N*-acetylglucosamine (GLcNAc) residues, forming the disaccharide unit *N*-acetylactosamine (Galß1-4GlcNAc) sequence with subsequent additions of further fucose, sialic acid, gal, GlcNAc and sulfate to form complex *N*-glycan branches [48]. In this pathway the oligomannose type *N*-glycan Man5 is converted initially to complex type *N*-glycans by mannosidases, the MGAT1, MGAT2 enzymes, and finally β-1,4-galactosyltransferase1 (B4GALT1). The substrates for these reactions are UDP-GlcNAc and UDP-galactose. In our previous studies we have noted dysregulation of expression of the precursor mannose pathway regulatory genes *MGAT1* and *MGAT3* in white blood cells of CG patients, decreased expression of the *B4GALT1* gene and overexpression of B4GALT1 activity in GALT deficient fibroblasts grown with galactose increased concentrations [32]. Thus, in this report the increased concentration of Man5 *N*-glycans with the presumed higher bioavailability of UDP-galactose *in vivo* in the higher galactose dietary intake group is of interest.

Mannose-5-glycans are known to hold a central dynamic position in the *N*-glycosylation pathway, influencing the fate of misfolded proteins, the ER-associated degradation system and the biological activity of glycoproteins. Dysregulation of the steps of oligomannose *N*-glycan synthesis is increasingly associated with complex diseases such as autoimmune diseases and cancer, with more extreme abnormalities presenting as the Congenital Disorders of Glycosylation [48], [49], [50].

Moreover, the consequences of dysregulation of the final step of Mannose-5-glycans incorporation into complex *N*-glycans are now well described in severe deficiency of B4GALT1 which causes a CDG condition characterised by brain embryonic structural dysplasias, global developmental delay and also systemic defects, including musculoskeletal disorders. In addition, aberrant gene expression of the *B4GALT1* gene variants have also been linked to autism spectrum disorders [51], [52].

## 5 Conclusion

In conclusion, our findings suggest that a moderate increase in dietary galactose intake (up to 500 mg/day) is well tolerated in our CG adult cohort and may improve CG adults' quality of life with improved dietary flexibility and food choices. While moderate relaxation of dietary restrictions is well-tolerated, higher galactose intake may lead to increased metabolic markers and possible subtle glycosylation abnormalities.

Additionally, novel biomarkers such as IgG *N*-glycans offer a promising tool for a more individualised personalised medicine approach, enabling more precise monitoring of dietary interventions. Integrating genetic factors, biochemical markers, and glycosylation profiles into individualised management plans may improve the quality of life for adult CG individuals.

Further long-term studies are needed to define the optimal balance between galactose restriction and liberalisation to maximize benefits while minimizing risks, given the lack of long-term correlation studies between mild -moderate galactose intake versus strict galactose restriction in CG.

Our results from this small study with reference to RBC Gal-1-P and related G0/G1 ratios suggest that a higher RBC Gal-1-P reference range may be more reasonable as a target reference range for CG adults on mild-moderate dietary galactose liberalisation. A reassessment of reference RBC Gal-1-P levels for adult CG patients on mild- moderately increased galactose intake (which could also apply to revised therapeutic goals for new therapies) is timely [12].

## 6 Limitations and future directions

The small sample size limits the generalizability of our findings. The retrospective nature of the dietary recall analysis may introduce a risk for bias in reporting precise dietary galactose intake. In this context, an estimated ‘range’ was used.

Future research with larger sample sizes and more precise monitoring of dietary intake would improve our understanding of how dietary galactose liberalisation impacts both glycosylation and clinical outcomes. While this study provides valuable data on moderate galactose liberalisation, the long-term effects of dietary flexibility in adults with CG remain unclear. Anecdotally, the individuals who relaxed diet reported an improved quality of life with more dietary choices. However, the retrospective nature of this study did not allow for a Quality-of-Life assessment.

Future studies could focus on longitudinal tracking of adult CG patients to determine the impact of continued dietary relaxation on cognitive, neurological, and reproductive outcomes. It is also of prime importance to consider glycan regulation and gene expression in the target organs such as brain and ovary.

## CRediT authorship contribution statement

**L.A. Shakerdi:** Writing – review & editing, Writing – original draft, Validation, Project administration, Methodology, Investigation, Formal analysis, Data curation, Conceptualization. **C. Newman Thacker:** Writing – review & editing, Writing – original draft, Validation, Methodology, Investigation, Formal analysis, Data curation. **K. Moore:** Writing – review & editing, Writing – original draft, Validation, Methodology, Investigation, Formal analysis, Data curation. **A. Sheerin:** Validation, Supervision, Project administration, Methodology, Investigation, Data curation, Conceptualization. **M. Noga:** Writing – review & editing, Validation, Formal analysis. **M.E. Rubio-Gozalbo:** Writing – review & editing, Formal analysis. **G.T. Berry:** Writing – review & editing. **J.J. O'Byrne:** Writing – review & editing, Supervision, Investigation. **R. Saldova:** Writing – review & editing, Writing – original draft, Visualization, Validation, Software, Resources, Project administration, Methodology, Investigation, Formal analysis, Data curation, Conceptualization. **E.P. Treacy:** Writing – review & editing, Writing – original draft, Validation, Supervision, Project administration, Methodology, Investigation, Funding acquisition, Formal analysis, Data curation, Conceptualization.

## Funding

This research did not receive any specific grant from funding agencies in the public, commercial, or not-for-profit sectors.

## Declaration of competing interest

The authors, declare that they have no conflict of interest. All procedures regarding the Galactosaemia patient records review were followed in accordance with the ethical standards of the responsible committee on human experimentation (institutional and national) and with the Helsinki Declaration of 1975, as revised in 2000.

Ethics approval was received by the Mater Misericordiae University Hospital Ethics Committee, IRB Ref: 1/378/1811.

## Appendix A Supplementary data

Supplementary material summary and Supplementary Figure S1. Image 1

Supplementary Table S1 Image 2
