# Supplementary material for: Galactose tolerance in adults with classical galactosaemia. Considering the gaps
Source: Mol Genet Metab Rep. 2026 Feb 26;46:101298. doi: 10.1016/j.ymgmr.2026.101298 (PMC12963900; doi:10.1016/j.ymgmr.2026.101298)
Supplement: Supplementary file 1 — Supplementary material summary and Supplementary Figure S1. [file mmc1.docx]

**Supporting data.**

**Supplementary Figures.**

**Supplementary Figure S1. Fucosylation, galactosylation and sialylation in the study groups.** Group 1: <200 mg gal/day, n = 9, Group 2: 200-500 mg, n = 12, Group 3: 500-1,000 mg (n = 6), Group 4: >1,000 mg. Sns violinplots were created using Seaborn package, 95% confidence interval with the mean value indicated. Fn= total fucosylated glycans, S0=asialylated glycans, S1=monosialylated glycans, S2=disialylated glycans, G0=agalactosylated glycans, G1=monogalactosylated glycans and G2=digalactosylated glycans.

**Supplementary Tables.**

**Table S1.** Significant differences among dietary groups
